# Thymoquinone played a protective role against tartrazine-induced hepatotoxicity

**DOI:** 10.22038/IJBMS.2022.67341.14763

**Published:** 2023-01

**Authors:** Nursena Demircigil, Mehmet Gul, Nurcan Gokturk, Elif Kayhan Kustepe, Harika Gozukara Bag, Mehmet Erman Erdemli

**Affiliations:** 1 Department of Medical Biochemistry, Medical Faculty, İnönü University, Malatya, Turkey; 2 Department of Histology and Embryology, Medical Faculty, İnönü University, Malatya, Turkey; 3 Department of Histology and Embryology, Medical Faculty, Turgut Ozal University, Malatya, Turkey; 4 Department of Biostatistics, Medical Faculty, Inonu University, Malatya, Turkey

**Keywords:** Hepatotoxicity, Inflammation, Oxidative stress, Rat, Tartrazine, Thymoquinone

## Abstract

**Objective(s):**

The current study, the first of its kind in the literature, aimed to observe the toxic effects of Tartrazine, a commonly used dyestuff in industries and foods, on the liver, and investigate whether this toxicity could be eliminated with thymoquinone coadministration.

**Materials and Methods:**

32 male Wistar albino rats were procured from İnönü University Experimental Animals Breeding and Research Center. The rats were randomly assigned to 4 equal groups: Control group, Thymoquinone group, Tartrazine group, and Thymoquinone + Tartrazine group. Rat liver tissue and blood samples were obtained and biochemical and histopathological examinations were conducted on the samples.

**Results:**

Tartrazine administration increased the oxidant (malondialdehyde and superoxide dismutase) and oxidative stress index parameters (total oxidant status) in the liver tissue and decreased the antioxidant parameters (glutathione, glutathione peroxidase, catalase, and total antioxidant status) leading to histopathological problems (hematoxylin-eosin staining and Caspase-3 immunoreactivity) and inflammation (tumor necrosis factor-α and interleukin-6) in the serum samples. Thymoquinone, on the other hand, improved antioxidant and anti-inflammatory effects.

**Conclusion:**

At this time and dose, thymoquinone has a protective effect against tartrazine hepatotoxicity. Thymoquinone can be used as a protective agent against tartrazine toxicity.

## Introduction

Tartrazine, which was authorized as the food additive E102 by the European Union, is a synthetic food dye employed in the food industry. It was first isolated from bitumen by the German chemist JH Ziegler in 1884 (1, 2). Tartrazine is an orange-yellow substance and could be in powder or granular form. It is produced from petroleum products. Tartrazine is a water-soluble and frequently preferred coloring used in several products due to its low cost. It is commonly used in puddings, bakery products, chips, ice cream, beverages, dairy products, meat and fish products, and confectionery among others. Tartrazine is not used only as a food dye, but also to color several personal care products (soap, moisturizer, toothpaste, shampoo, and hair care products) and certain drugs (3, 4). 

It was reported that the primary metabolism mechanism for tartrazine in nutrients is bacteria in humans, rats, and rabbits after oral intake. Sulfanilic acid and aminopyrazolone are the main metabolites of tartrazine (5, 6) It was suggested that the azo dyes in tartrazine could have mutagenic, carcinogenic, and toxic effects due to the reduction of the biotransformation effect of the azo bond (7-10). 

Thymoquinone, which is the main bioactive component in *Nigella sativa* essential oil, is a volatile monoterpene quinone with a molecular weight of 164.2 g/mol in the form of dark yellow crystals (11). It was reported that thymoquinone has antioxidant, immunomodulatory, hepatoprotective, anticarcinogen, gastroprotective, anti-inflammatory, hypoglycemic antimicrobial, nephroprotective, antidiabetic, neuroprotective, hypolipidemic, and antihistaminic effects, and affects the heart and respiratory system diseases and apoptosis. It was also reported that it could be a natural remedy for autoimmune diseases (12-16). 

In the current study, the possible adverse effects of tartrazine on the liver, and whether thymoquinone could alleviate the toxic effect of tartrazine, if it exists, were analyzed for the first time in the literature.

## Materials and Methods

***Procurement and care of the rats***

Our study was conducted with 32 albino male Wistar rats procured from İnönü University Experimental Animal Breeding and Research Center. Rats were fed *ad libitum* with standard pellet feed and tap water throughout the experiments.

***Experimental groups***

Group 1: (Control group): Corn oil administration.

Group 2: (Tartrazine group): 100 mg/kg/day tartrazine was administered (Sigma-Aldrich-1934-21-0, St. Louis, USA) (17).

Group 3: (Thymoquinone group): 50 mg/kg/day thymoquinone was administered (Sigma-Aldrich-490-91-5, St. Louis, USA) (18).

Group 4: (Tartrazine + Thymoquinone group): 100 mg/kg/day tartrazine+ 50 mg/kg/day thymoquinone were administered.

Tartrazine was applied by gavage after it was dissolved in physiological saline and thymoquinone was dissolved in corn oil and 1 ml/kg/day solution was administered to each rat. The solutions were administered at the same time every day for 21 days.

***Collection of the samples and preparations***

After the experiments, the abdominal region of the rats was opened under anesthesia (xylazine and ketamine) with the protocol prescribed by the ethics committee, and blood samples were obtained from the heart tissue and transferred into adequate tubes. Liver tissues were incised and washed with physiological saline to remove the excess blood. Certain liver tissues were placed in sterile containers and quickly frozen to -80 °C for biochemical analysis. The rest of the liver tissues were placed in containers that included 10% formol solution for histopathological analyses.

***Biochemical analysis***

Liver tissues were removed from the -80 °C freezer and quickly weighed before thawing. Phosphate buffer that equaled 9 times the tissue weight was added. They were homogenized at 15000 rpm for 1 min at +4 °C (IKA, Germany). Malondialdehyde (MDA) levels were determined with these homogenates. The tissue homogenates were centrifuged at 4000 rpm for 25 min at +4 °C to obtain the supernatants. Glutathione (GSH), superoxide dismutase (SOD), glutathione peroxidase (GSH-Px), catalase (CAT), total oxidant status (TOS), total antioxidant status (TAS), oxidative stress index (OSI), and protein levels were determined on these supernatants. Blood samples were centrifuged at 600 g, 4 °C for 20 min to obtain the serum, and the serum was used to determine tumor necrosis factor-alpha (Tnf-α) and interleukin-6 (IL-6).

***MDA analysis***

Tissue homogenate 0.25 ml tissue homogenate was mixed with 1.5 ml 1% H3PO4 and 0.5 ml 0.6% thiobarbituric acid. The product was heated to 30 °C to completely dissolve the TBA. The mixture was kept at 100 ºC for 45 min. Then, the formation of the red color was observed, and then the tubes were cooled in tap water and 2 ml n-butanol was added to each sample. Each tube was vortexed for 5 min. Then, the samples were centrifuged for 25 min at 5000 rpm, 25 °C. 25 µl supernatant (n-butanol phase) was carefully collected and transferred to individual quartz microplate wells. Readings were conducted at 535 nm. The findings are presented as nanomole/gram wet tissue (19).

***GSH analysis***

The supernatant was deproteinized with tricarboxylic acid (TCA). 125 µl TCA was added to the 125 µl sample. After the mixture was vortexed, it was centrifuged for 20 min at 5000 rpm and +4 °C to obtain the protein-free supernatant. 29 µl deproteinized sample, 29 µl DTNB, and 235 µl Na_2_HPO_4_ were added to each microplate well separately, gently vortexed, and kept for 5 min. Readings were conducted at 410 nm within 5 min. The findings are presented as nanomole/gram wet tissue (20).

***SOD enzyme activity***

SOD enzyme activity is determined with measurement of the intensity of this color formed by the reduction of NBT by superoxide radicals (xanthine + xanthine oxidase system) with a spectrophotometer. The reduction forms a blue-colored formazan with a maximum absorbance of 560 nm. In the absence of an enzyme, this reduction is maximal, and a dark blue color is observed. In the presence of SOD, the enzyme converts the superoxide radical to hydrogen peroxide; thus, the reduction of NBT is lower and no blue formazan is observed or the intensity of the color is quite light. SOD activity was calculated based on the absorbance of the blue-colored formazan at 560 nm. The results are presented as U/mg protein (21).

***CAT enzyme activity***

Hydrogen peroxide (H_2_O_2_) is a substance that provides absorbance in the UV spectrum and its maximum absorbance wavelength is 240 nm. A decrease in absorbance is observed at 240 nm due to the breakdown of the added hydrogen peroxide into water and oxygen by catalase. This decrease in absorbance was recorded for 1 min via kinetic readings and the enzyme activity was measured. The enzyme activity is presented as K/g protein (22).

***GSH-px activity***

GSH-Px is an enzyme that catalyzes the conversion of hydrogen peroxide to water by reduced glutathione. After the reaction, reduced glutathione is converted to the oxidized form. For the conversion of another hydrogen peroxide into water, the oxidized glutathione should be converted back to the reduced form. This occurs in the presence of reduced NADP and reduced glutathione in the medium. Then, reduced NADP is converted into oxidized NADP, while oxidized glutathione is converted into reduced glutathione. The maximum absorbance of the reduced NADP is observed at 340 nm. The absorbance drops at 340 nm as glutathione reductase catalysis continues, since the reduced NADP is not converted into the oxidized form. GSH-Px activity is calculated by recording the decrease in absorbance for 3 min. The findings are presented as U/mg protein (23).

***Protein analysis***

The tissue protein content is required to calculate enzyme activities. The Lowry method was used to determine the tissue protein content. The absorbance of the resulting color was measured at 660 nm in the spectrophotometer to determine the protein content (24).

***TAS analysis***

Rel Assay brand kit (Rel Assay Diagnostics, Gaziantep Turkey) was used to determine the TAS. The measurement is based on the discoloration of the antioxidant molecules. Sequential steps were conducted based on the kit instructions. TAS was determined with the measurement of the absorbance at 660 nm in the device set for 37 °C as specified in the kit. The findings are presented as mmol Trolox Equiv/l (25).

***TOS analysis***

TOS Rel Assay brand kit (Rel Assay Diagnostics, Gaziantep, Turkey) was employed to determine the TOS. Ferric ions form a colored chromogenic solution when the oxidants in the sample convert the ferrous ion chelator complexes into ferric ions. TOS is determined with the measurement of the absorbance of the colored complex at 530 nm, 25 °C as specified in the kit. The findings are presented as μmol H_2_O_2 _equiv/l (26).

***OSI analysis***

The OSI was calculated with the formula OSI (arbitrary unit) = TOS (μmol H_2_O_2_ eqv/L) / TAS (mmol Trolox eqv/L) X 10. Results are presented as arbitrary units (AU).

***TNF- α and IL-6 analysis***

Bioassay Technology Laboratory ELISA Kit was employed to determine the serum interleukin-6 and TNF-α levels. All reagents were at room temperature before the tests, and the tests were performed at room temperature. The sample and ELISA reagent were transferred to the wells, and these were incubated for 1 hr at 37 °C. Then, they were washed five times in the microplate washer with the lavage solution. Substrate solutions A and B were added and incubated at 37 °C for 10 min. Then, the stop solution was added, and the color change was observed. The readings were conducted within ten min. The findings are reported as ng/l.

***Histopathological analysis***

For histopathological analysis, liver tissue samples were fixed in 10% formaldehyde for 48 hr. Then, liver tissue samples were dehydrated through an incremental ethanol series (50%, 70%, 80%, 96%, and absolute). The liver tissue samples were then transparentized through the xylene series and embedded in paraffin blocks after they were passed through the melted paraffin series at 62 °C for infiltration. 6 µm thick sections were obtained from the paraffin blocks with a microtome and placed on slides (27). They were stained with hematoxylin-eosin (H-E) and anti-caspase-3 [Cleaved-CASP3p17 (D175) Polyclonal Antibody] (Elabscience, Texas, USA) for immunohistochemical (IHC) analysis. Stained sections were examined with Nikon Eclipse Ni-U light microscope, Nikon DS-Fi3 microscope camera, and Nikon NIS-Elements Documentation 5.02 image analysis program (Nikon Corporation, Tokyo, Japan).

Histopathological changes in the liver sections stained with hematoxylin-eosin (inflammatory cell infiltration, necrosis, periportal edema, and vascular congestion) were scored between 0 and 3 (0; absent, 1; mild-rare, 2; moderate, 3; severe- common), where the maximum histopathological score was 12.

***Immunohistochemistry analysis***

Cleaved caspase-3 expression was considered an indicator of apoptosis in liver tissue. Sections stained for immunohistochemical analysis were placed on polylysine-coated slides. Sections were initially deparaffinized. Then, they were heat-treated in a Retriever 2100 (Aptum, Southampton, UK) for 15 min with citrate buffer with a pH of 7.6 (Thermo Scientific, Fremont, CA, USA) for retrieval of the antigens. After the sections were cooled to room temperature for 20 min, they were first washed with distilled water and then with phosphate-buffered saline (PBS) for 1–2 min. Sections were drawn using a hydrophobic pen and lined up on the platform and treated with 3% hydrogen peroxide for 10 min to inhibit endogenous peroxidase activity, then washed with PBS. Sections were incubated with protein-V blocking reagent (Thermo Scientific) for 5 min.

Sections were incubated with 1:200 diluted primary rabbit polyclonal cleaved caspase-3 antibody (Cleaved-CASP3p17 (D175) Polyclonal Antibody) (Elabscience, Texas, USA) for 1 hr, then rinsed in PBS, incubated with biotinylated goat anti-polyvalent secondary antibody for 10 min, and transferred into PBS. It was then incubated with streptavidin peroxidase (HRP) for 10 min and transferred into PBS. The polyvalent HRP kit (Thermo Scientific) was employed in compliance with the manufacturer’s instructions. Finally, sections were treated with chromogen (AEC; Thermo Scientific) + substrate buffer (AEC) (Thermo Scientific) for a maximum of 15 min. After they were washed with PBS and distilled water, counterstaining was conducted with Mayer’s hematoxylin for 1 min. Sections were rinsed in tap water and then distilled water and covered with a water-based sealer and coverslip (Thermo Scientific, Cheshire, UK).

The caspase-3 immunoreactivity H score (H Score=Pi (i+1), Pi, is the percentage of stained cells in each density category (0–100%) in sections stained with anti-caspase-3 in the immunohistochemical method, and i denotes weak (i = 1), moderate (i = 2), or strong staining (i = 3) (28).

***Statistical method***

Numerical data were summarized with median, minimum, and maximum values. The Kruskal-Wallis test was used for independent group comparisons and the Friedman test was used for dependent group comparisons. After both universal tests and pairwise comparisons were made with the Conover method. The significance level was accepted as 0.05 in all tests.

## Results

***Biochemical***

No significant difference was determined between the changes in rat weight based on the groups. Weight gain was observed in rats in all groups each week (Table 1).

It was determined that the oxidative stress parameters MDA and SOD levels increased and GSH, GSH-Px, and CAT levels decreased in the liver tissue of the Tartrazine group rats when compared with all other groups. TOS increased and TAS decreased in the liver tissue of the Tartrazine group rats when compared with all other groups. Tartrazine led to an increase in Tnf-α and IL-6, the inflammatory agents, in rat serum when compared with all other groups. Thymoquinone administration led to an increase in CAT, GSH, and GSH-Px, and a decrease in MDA and SOD levels. Thymoquinone administration increased the TAS levels and decreased the TOS in rat liver tissue when compared with all other groups. Serum Tnf-α and IL-6 levels decreased with thymoquinone administration when compared with all other groups. Tartrazine + Thymoquinone administration improved oxidative stress parameters, OSI markers, and inflammation factors when compared with the tartrazine group (Tables 2, 3, and 4).

***Histopathology***

**Figure 1:**
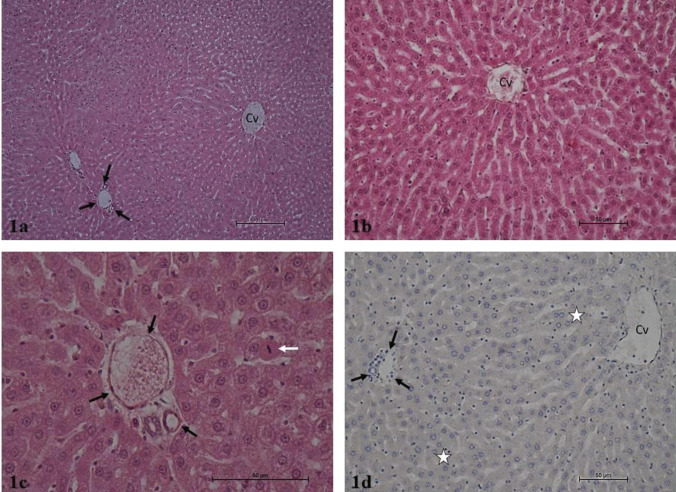
a. Control group. Liver parenchyma in normal histological structure. Central vein (Cv), portal region (arrows). H-E, x10.b. Control group. Liver parenchyma in normal histological structure. Central vein (Cv), portal region (arrows). H-E, x40. c. Control group. Liver parenchyma in normal histological structure. Mitosis figure (white arrow), portal region (black arrows). H-E, x40. d. Control group. Liver parenchyma, caspase-3 immunoreactivity negative (asterisk), central vein (Cv), portal region (arrows). H-E, x20

**Figure 2:**
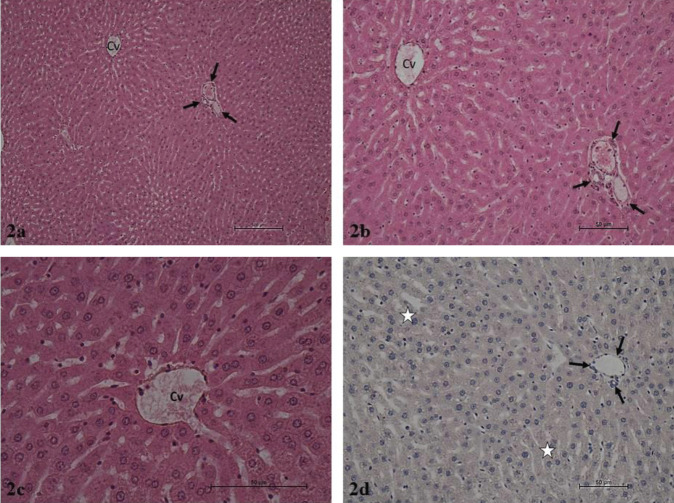
a. Thymoquinone group. Liver parenchyma in normal histological structure. Central vein (Cv), portal region (arrows). H-E, x10.b. Thymoquinone group. Liver parenchyma in normal histological structure. Central vein (Cv), portal region (arrows). H-E, x20. c. Thymoquinone group. Liver parenchyma in normal histological structure. Central vein (Cv). H-E, x40. d. Thymoquinone group. Liver parenchyma, negative caspase-3 immunoreactivity (asterisk), portal area (arrows). IHC, x20

**Figure 3:**
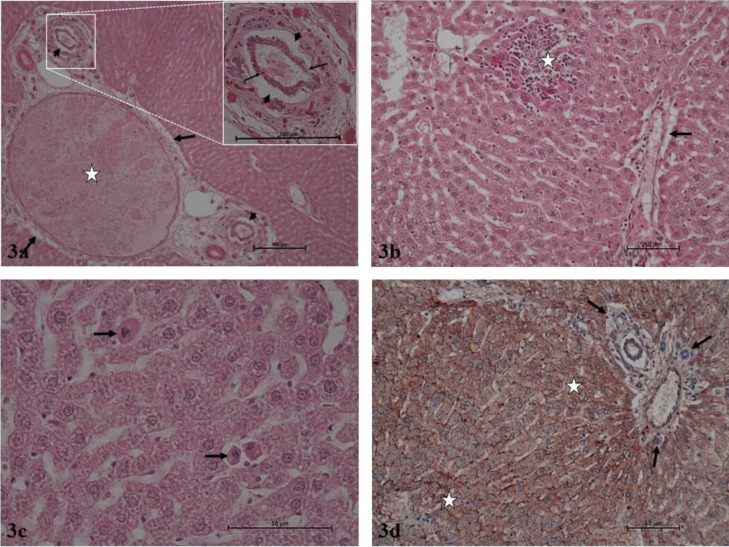
a. Tartrazine group. Large image. Portal vein lumen congestion (asterisk), periportal edema (bold arrows), periductal edema (arrowhead). H-E, x10. Small image. Periductal edema (arrowheads), cholangiocyte damage, and degeneration (arrows). H-E, x40. b. Tartrazine group. Necrosis and inflammatory cell infiltration (asterisk) in the liver parenchyma. Periportal edema (arrow). H-E, x20. c. Tartrazine group. Apoptotic hepatocytes in liver parenchyma (arrows). H-E, x40. d. Tartrazine group. Liver parenchyma, strong positive caspase-3 immunoreactivity (asterisk), portal region (arrows). H-E, x20

**Figure 4:**
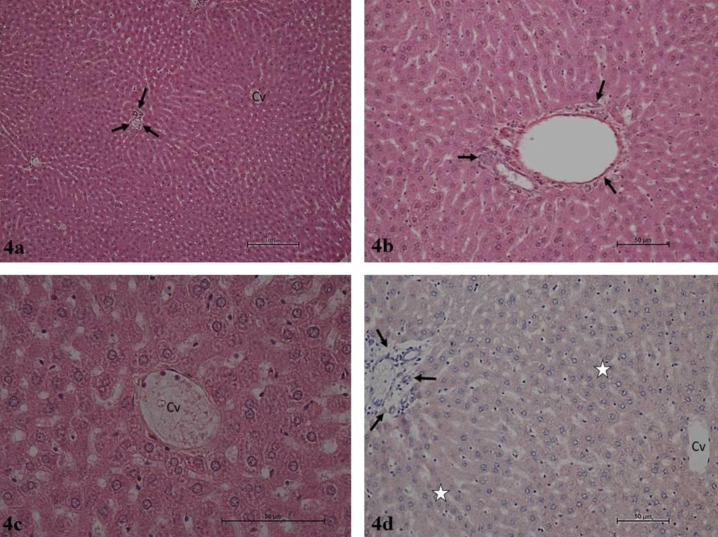
a. Tartrazine + Thymoquinone group. Liver parenchyma in normal histological structure. Central vein (Cv), portal region (arrows). H-E, x10.b. Tartrazine + Thymoquinone group. Liver parenchyma in normal histological structure. Portal region (arrows). H-E, x20. c. Tartrazine + Thymoquinone group. Liver parenchyma in normal histological structure. Central vein (Cv). H-E, x40. d. Tartrazine + Thymoquinone group. Liver parenchyma, weak positive caspase-3 immunoreactivity (asterisk), portal area (arrows). H-E, x20

It was determined that the hepatocyte cords, sinusoids, central vein, and portal areas in liver parenchyma exhibited normal histology after hematoxylin-eosin staining in the Control and Thymoquinone groups. Liver lobules presented the usual structure, including radially organized hepatocyte cords and sinusoids around the central vein. The portal triad connective tissue and related vascular structures and bile ducts were open and presented normal histological structures (Figures 1 and 2). The hematoxylin-eosin-stained liver sections in the Tartrazine group exhibited necrosis of various lengths in the liver parenchyma. Inflammatory cell infiltration was observed in the regions of parenchymal necrosis and portal and periportal regions. Apoptotic hepatocytes with dark eosinophilic cytoplasm and heterochromatic pycnotic nuclei were identified in the parenchyma. Congestion was noted in vena porta branches in the portal regions. The mall distance between the portal region connective tissue and peripheral hepatocytes was significantly larger due to periportal edema. Furthermore, periductal edema was observed around the bile ducts in the portal region. Damaged and degenerated cholangiocytes were noted in the bile duct (Figure 3). In the hematoxylin-eosin-stained liver sections of the rats in the Tartrazine + Thymoquinone group, rare focal necrosis, minimal inflammatory cell infiltration, and periportal edema were observed in the parenchyma. However, the extent of these damages was significantly reduced when compared with the Tartrazine group (Figure 4).

Caspase-3 immunoreactivity was similarly weak and minimal in the immunohistochemical anti-caspase-3 staining administration regions in the control and Thymoquinone groups. In these groups, the caspase-3 immunoreactivity H score was 1. Intense and prevalent caspase-3 immunoreactivity was observed in the same regions in the Tartrazine group. Tartrazine group caspase-3 immunoreactivity H score was 180. The caspase-3 immunoreactivity staining in the regions in the Tartrazine + Thymoquinone group was generally low to moderate intensity. The Tartrazine group exhibited a caspase-3 immunoreactivity H score of 60 (Table 5).

## Discussion

Currently, food dyes are frequently used to improve the attractiveness and aesthetics of the food products such as confectionery products. Due to the advances in the food industry and consumer trends that prefer packaged food items, it became almost impossible to avoid food dyes. It became very difficult to prevent the use of these substances, which could induce hyperactivity, allergic reactions, and asthma, especially among children. Tartrazine, which is a common food dye, could have toxic effects, and induce oxidative stress (29-32), which could lead to neurotoxicity, nephrotoxicity, and hepatotoxicity (17, 33). Amin *et al*. investigated the effects of tartrazine and carmoisine on biochemical parameters associated with kidney and liver functions and oxidative stress markers in young male rats and administered two different doses (500 mg/kg/BW and 100 mg/kg/BW) of tartrazine to rats orally for thirty days. They reported that SOD, GSH, and CAT levels decreased, and MDA levels increased significantly in the high-dose group when compared with the control (8). In a study where oxidative stress and hepatotoxicity induced by tartrazine were analyzed in male rats, 7.5 mg/kg tartrazine was administered to rats for 90 days. The analysis of the rat liver tissues after 90 days revealed MDA and total protein levels increased and SOD, GPX, GSH, and CAT levels decreased significantly in the tartrazine group when compared with the control group that was fed a normal diet. Furthermore, they reported histopathological changes in liver tissue (34). In a study conducted by Velioğlu *et al*. 500 mg/kg/day, tartrazine was administered to rats orally for 21 days. The comparison of the tartrazine and control groups revealed that SOD, MDA, and TOS levels significantly increased, and CAT, TAS, and GSH levels decreased in the liver tissues of the tartrazine group, leading to the histopathological damage in the liver tissue (10). Abd-Elhakim *et al*. conducted a study on rats and administered 75 mg/kg/BW tartrazine orally for 90 days. Significant increases were observed in mRNA levels and immunohistochemical localization of collagen 1-a, TGFβ-1, and caspase-3. Furthermore, significant increases were observed in AST, ALP, MDA, creatinine, and urea levels, and significant decreases were observed in SOD, CAT, and GSH enzyme levels in the kidney and liver tissues when compared with the control group. In the histological examination, hepatocytes, apoptotic hepatocytes, and periportal fibrosis with tubular necrosis were observed (35).

Thymoquinone is a phenolic compound found in the *N. Sativa* plant seed oil, and it is traditionally used to treat several diseases due to its high antioxidant properties. *In vivo *and* in vitro* studies suggested that thymoquinone could have anti-inflammatory, antioxidant antimicrobial, and anticarcinogenic properties (36, 14). Erdemli *et al*. investigated the changes in liver tissues induced by thymoquinone administration against 2,3,7,8-tetrachlorodibenzo-p-dioxin (TCDD)-induced hepatotoxicity; 50 rats were divided into five groups, and the administration was maintained for thirty days. The study was conducted on corn oil, TCDD, thymoquinone, and TCDD + Thymoquinone groups. They reported that TCDD administration led to histopathological changes such as thickening of the Glisson’s capsule in the liver, intracytoplasmic vacuolization, sinusoidal expansion, vascular and sinusoidal occlusion, and inflammatory cell infiltration in hepatocytes, based on biochemical and histopathological analyses. CAT, GSH, SOD, and TAS levels increased, and MDA, TOS, ALT, AST, and ALP levels decreased in the thymoquinone treatment group when compared with all other groups (37). In a 28-day study where the protective properties of thymoquinone against arsenic-induced hepatotoxicity were investigated in rats, it was reported that SOD, CAT, GSH-Px, and GSH levels significantly decreased after arsenic exposure, and these parameters significantly improved after thymoquinone treatment (38). In a study, the potential protective properties of thymoquinone were investigated in Pb-induced liver damage. Adult male rats in the Pb group (2000 ppm Pb acetate via drinking water) were administered 5 mg/kg/day thymoquinone for 5 weeks. The findings demonstrated that Pb exposure increased hepatic Pb content, damaged hepatic histological structure (necrotic foci, hepatic filament disorder, hypertrophic hepatocytes, cytoplasmic vacuolization, cytoplasmic loss, chromatin condensation, mononuclear cell infiltration, congestion, and centrilobular swelling) and altered liver functions. They also reported that Pb administration reduced total antioxidant status and increased lipid peroxidation in the liver. It was reported that the thymoquinone supplement significantly ameliorated the adverse effects induced by Pb (39). In a study where hepatorenal protection mechanisms of thymoquinone were investigated in methotrexate-induced rat toxicity, the authors administered oral thymoquinone (10 mg/kg) for 10 days. The comparison of the methotrexate and thymoquinone + methotrexate groups revealed that the methotrexate-induced decreases in the liver and kidney GSH and CAT levels approached control group levels, and the increased MDA and TNF-𝛼 levels decreased (40). In the current study, the analysis of the efficacy of thymoquinone against food dye tartrazine toxicity in the liver tissue revealed similar findings when compared with other reports in histological, immunohistochemical, and biochemical analyses. The findings demonstrated that Tartrazine led to hepatotoxicity via oxidative stress and inflammation, while Thymoquinone minimized Tartrazine-induced hepatotoxicity due to strong antioxidant and anti-inflammatory properties.

**Table 1:** Weekly weight changes of rats

**Groups**	**1st Week**	**2nd Week**	**3rd** **Week**
**Control**	295.5 (280-307) ^a^	315.5 (293-342)^ b^	328.5 (302-352) ^c^
**Thymoquinone**	274 (249-290) ^a^	295 (267-315) ^b^	303 (275-320) ^c^
**Tartrazine**	250.5 (232-271) ^a^	271 (243-282) ^b^	281 (252-290) ^c^
**Thymoquinone +** **Tartrazine**	268.5 (241-298) ^a^	274 (243-314) ^b^	288 (246-319) ^c^
*P*	<0.001	<0.001	<0.001

Note: Data are expressed as median (minimum-maximum). There is a statistically significant difference between groups marked with different letters

**Table 2.** Oxidant–anti-oxidant parameters of rats

**Groups**	**MDA ** (nmol/gwt)	**GSH ** (nmol/gwt)	**SOD ** (U/mg protein)	**CAT ** (K/mg protein)	**GSH-Px ** (U/mg protein)
**Control**	116.9 (108.8-126.4) ^a^	1089 (1008-1351) ^a^	1.9 (1.2-3.1) ^a^	48.7 (38.2-60.2) ^a^	262 (246.307) ^a^
**Thymoquinone**	102 (87.72-117.6) ^b^	1349 (1062-1972) ^b^	0.9 (0.4-2.2) ^b^	65.4 (62.3-67.9) ^b^	290 (262-325) ^b^
**Tartrazine**	132 (121-138.8) ^c^	885 (809-947) ^c^	2.9 (2.3-4) ^c^	30.3 (25.-38.9) ^c^	230 (204-262) ^c^
**Thymoquinone ** **+** **Tartrazine**	112.2 (95.2-129.8) ^a,b^	1086 (932-1198) ^a^	1.5 (0.7-2.2) ^b^	37.8 (34.5-47.2) ^d^	242 (213-270) ^d^
*P*	<0.001	<0.001	<0.001	<0.001	<0.001

Note: Data are expressed as median (minimum-maximum). There is a statistically significant difference between groups marked with different letters

MDA, malondialdehyde; GSH, reduced glutathione; SOD, superoxide dismutase; CAT, catalase; GSH-Px, glutathione peroxidase, gwt, gram wet tissue

**Table 3:** Oxidative stress index parameters of rats

**Groups**	**TOS ** (µmol H_2_O_2_ Eqv/l)	**TAS** (mmol Trolox Eqv/l)	**OSI** (AU)
**Control**	17.8 (16.4-21.8) ^a^	2.25 (2.1-2.27) ^a^	81.3 (75-96.2) ^a^
**Thymoquinone**	14.9 (13-19) ^b^	4.07 (3.86-4.98) ^b^	37.8 (26.7-39.2) ^b^
**Tartrazine**	28.2 (25.9-29.4) ^c^	1.99 (1.82-2.08) ^c^	140 (124.9-162) ^c^
**Thymoquinone +** **Tartrazine**	16.8 (15.1-21.2) ^a^	2.13 (1.84-2.31) ^d^	82.2 (74.6-95.4) ^a^
*P*	<0.001	<0.001	<0.001

Note: Data are expressed as median (minimum-maximum). There is a statistically significant difference between groups marked with different letters. Abbreviations: TAS, total antioxidant status; TOS, total oxidant status; OSI, oxidative stress index

**Table 4:** Inflammation parameters of rats

**Groups**	**TNF-α** (ng/L)	**IL-6** (ng/L)
C**ontrol**	82.6 (69.4-112.5) ^a^	63.9 (46.4-79.7) ^a^
**Thymoquinone**	76.4 (74.1-96) ^b^	57.3 (41.1-69.1) ^b^
**Tartrazine**	104.5 (78.8-116) ^c^	83.6 (67.8-89.9) ^c^
**Thymoquinone ** **+** **Tartrazine**	89 (73.1-110.5) ^d^	73.5 (57.2-86) ^d^
*P*	<0.001	<0.001

Note: Data are expressed as median (minimum-maximum). There is a statistically significant difference between groups marked with different letters. Abbreviations: tumor necrosis factor-alpha, (Tnf-α); and interleukin-6, (IL-6)

**Table 5:** Histopathology and Caspase-3 damage scores of rats

**Groups**	**Inflammatory** **cell infiltration**		**Necrosis**	**Periportal** **edema**	**Vascular** **congestion**	**Total** **damage score**	**Caspase-3** **damage score**
**Control**	0 (0-0) ^a^	0 (0-0) ^a^		0^a^ (0-0) ^a^	0^a^ (0-0) ^a^	0 (0-0) ^a^	1 (1-10)^ a^
**Thymoquinone**	0 (0-0) ^a^	0 (0-0) ^a^		0 (0-0) ^a^	0 (0-0) ^a^	0 (0-0) ^a^	1 (1-10) ^a^
**Tartrazine**	1 (1-2)^ b^	1 (1-2) ^b^		1 (1-2) ^b^	2 (1-2) ^b^	5 (4-7)^ b^	180 (160-240) ^b^
**Thymoquinone ** **+** **Tartrazine**	0 (0-1) ^c^	0 (0-1) ^c^		1.5 (0.7-2.2) ^c^	0.5 (0-1) ^c^	1 (1-3) ^c^	60 (50-80) ^c^
*P*	<0.001	<0.001		<0.001	<0.001	<0.001	<0.001

## Conclusion

Exposure to food coloring substances has increased with the prevalence of take-home food products. Thus, consumption of the common substances in these food items such as tartrazine could not be avoided. To eliminate the toxic effects of tartrazine or at least to minimize these hepatoxic effects, we recommend daily consumption of *N. sativa* and its active component, thymoquinone, when possible.

## Authors’ Contributions

ND and NG studied biochemical analysis; MEE, ND, and NG designed the study and collected the tissues; MG and EKK performed the histological examination of the liver tissues; HGB calculated the biochemical and histological results; MEE was a major contributor in writing the manuscript. 

## Ethical Approval and Informed Consent

The study was approved by the Experimental Animals Ethics Committee of Inonu University, Faculty of Medicine (Protocol No: 2020/17-4). 

## Conflicts of Interest

The authors declare no competing interests.
